# Ketone body 3-hydroxybutyrate enhances adipocyte function

**DOI:** 10.1038/s41598-022-14268-w

**Published:** 2022-06-16

**Authors:** Shigeki Nishitani, Atsunori Fukuhara, Issei Tomita, Shinji Kume, Jihoon Shin, Yosuke Okuno, Michio Otsuki, Hiroshi Maegawa, Iichiro Shimomura

**Affiliations:** 1 Departments of Metabolic Medicine, Osaka University Graduate School of Medicine, Suita, Osaka Japan; 2 Departments of Lifestyle Medicine, Osaka University Graduate School of Medicine, Suita, Osaka Japan; 3 Departments of Adipose Management, Osaka University Graduate School of Medicine, Suita, Osaka 565–0871 Japan; 4 Departments of Diabetes Care Medicine, Osaka University Graduate School of Medicine, Suita, Osaka Japan; 5 Department of Medicine, Shiga University of Medical Science, Tsukinowa-cho, Seta, Otsu, Shiga Japan

**Keywords:** Nutrient signalling, Endocrine system and metabolic diseases

## Abstract

Ketone bodies, including 3HBA, are endogenous products of fatty acid oxidation, and Hmgcs2 is the first rate-limiting enzyme of ketogenesis. From database analysis and in vivo and in vitro experiments, we found that adipose tissue and adipocytes express Hmgcs2, and that adipocytes produce and secrete 3HBA. Treatment with 3HBA enhanced the gene expression levels of the antioxidative stress factors, PPARγ, and lipogenic factors in adipose tissue in vivo and in adipocytes in vitro, accompanied by reduced ROS levels. Knockdown of endogenous Hmgcs2 in adipocytes markedly decreased 3HBA levels in adipocytes and decreased the gene expression levels of the antioxidative stress factors, PPARγ, and lipogenic factors with increased ROS levels. Conversely, overexpression of Hmgcs2 in adipocytes increased 3HBA secretion from adipocytes and enhanced the gene expression levels of the antioxidative stress factors, PPARγ, and lipogenic factors. These results demonstrate that 3HBA plays significant roles in enhancing the physiological function of adipocytes.

## Introduction

The ketone body, including 3-hydroxybutyric acid (3HBA), is an endogenous product of fat metabolism, and a vital alternative metabolic fuel source for all domains of life, eukarya, bacteria, and archaea^1^. In mammals, 3HBA is predominantly synthesized in the liver from acetyl-CoA derived from β-oxidation of fatty acids^2^, transported to extrahepatic tissues, taken up in peripheral tissues by monocarboxylate transporter (MCT), and oxidized for ATP production^3^. 3HBA becomes an important energy source within extrahepatic tissues in various physiological states, such as postexercise, fasting, starvation, pregnancy, the neonatal period, and adherence to low-carbohydrate diets. Circulating total ketone body concentrations in healthy adult humans normally exhibit circadian oscillations between approximately 0.1 and 0.25 mM, rise to 1 mM after prolonged exercise or 24 h of fasting, and can accumulate to as high as 20 mM in pathological states such as diabetic ketoacidosis.

3-Hydroxy-3-methylglutaryl-CoA synthetase 2 (Hmgcs2) is the first rate-limiting enzyme in the production of ketone bodies. Several reports have revealed the extrahepatic expression of hmgcs2, and the production of ketone bodies. Retinal pigment epithelial (RPE) cells express particularly high levels of hmgcs2 and metabolize fatty acids to produce 3HBA, which is transported to the retina for use as a metabolic substrate^4^. Astrocyte cell lines produce ketone bodies by treatment with lauric acid^5^. In the small intestine, Hmgcs2 expression is highly enriched in self-renewing Lgr5 + intestinal stem cells (ISCs), which produce ketone bodies to regulate intestinal stemness^6^. When artificially induced by retroviral overexpression of PRDM16, beige adipocytes produce and secrete 3HBA with upregulation of Hmgcs2^7^, however there were no reports about ketogenesis in white adipocytes.

Although 3HBA has been an energy source, recent evidence demonstrates that 3HBA is also a signaling metabolite^8^. 3HBA inhibits class I HDACs, which increases histone acetylation and thereby induces the expression of genes. Regarding the small intestine, 3HBA mediates intestinal stem cell homeostasis through enhancement of Notch signaling by inhibition of HDAC activity^6^. 3HBA suppresses oxidative stress by inducing the expression of antioxidative factors, such as forkhead box O3 (Foxo3), metallothionein 2 (Mt2), MnSOD and catalase, with increasing acetylation of lysine residues in histone H3 by inhibition of HDAC^9^. In cardiomyocytes, 3HBA is produced as a compensatory response against oxidative stress in heart failure through inhibition of HDAC^10^. Moreover, histone lysine residues are subject to direct epigenetic modification by 3HBA, which is associated with active gene expression^11^. Related to this study, we reported that 3HBA induces β-hydroxybutyrylation of histone H3 at lysine 9 and upregulation of adiponectin in 3T3-L1 adipocytes independent of their acetylation or methylation^12^.

In the present study, we identified that Hmgcs2 is an upregulated gene in adipose tissues of fasted mice and is expressed in differentiated 3T3-L1 adipocytes. In addition, treatment with 3HBA or overexpression of the Hmgcs2 gene enhanced, but silencing of the Hmgcs2 gene impaired, adipocyte function to regulate the expression of antioxidative stress factors, PPARγ, lipogenic genes, and insulin signaling.

## Results

### Ketone body 3HBA was physiologically regulated

**Figure 1:**
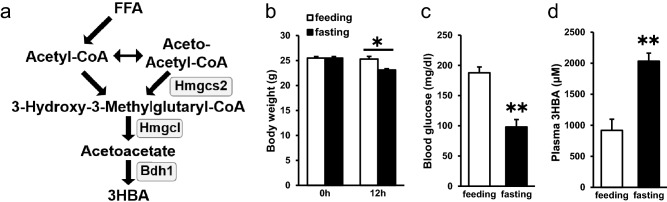
Ketone body 3HBA was physiologically regulated. (**a**) Schematic representation of ketone body metabolism. FFA, free fatty acid; Hmgcs2, 3-hydroxy-3-methylglutaryl-CoA synthetase; Hmgcl, 3-hydroxy-3-methylglutaryl-CoA lyase; Bdh1, β-hydroxybutyrate dehydrogenase; 3HBA, 3-hydroxybutyric acid. (**b**) Body weight of C57BL/6 J mice in the feeding and fasting groups at the beginning (0 h) and end (12 h) of the experiment. n = 3. (**c** and **d**) Blood glucose (**c**) and plasma 3HBA concentrations (**d**) of C57BL/6 J mice after 12 h of feeding and fasting. n = 3. Data are mean ± SEM. *p < 0.05, **p < 0.01.

Conventionally, production of ketone body, i.e., Ketogenesis occurs primarily in the mitochondria of liver cells. In short, Hmgcs2 converts acetyl-CoA derived from β-oxidation of free fatty acids (FFAs) into 3-hydroxy-3-methylglutaryl-CoA, which is catalyzed into acetoacetate by 3-hydroxy-3-methylglutaryl-CoA lyase (Hmgcl). β-Hydroxybutyrate dehydrogenase (Bdh1) then converts acetoacetate to 3HBA (Fig. 1a).

To validate the physiological regulation of 3HBA, wild-type C57BL/6 male mice were subjected to feeding or fasting conditions for 12 h, and then, blood samples and adipose tissues were collected. Body weight and blood glucose were decreased in the fasting group compared with the feeding group (Fig. 1b and c), whereas the plasma 3HBA concentration was higher in the fasting group than in the feeding group (Fig. 1d), as previously reported^13^.

### Systemic Hmgcs2 knockout mice showed decreased circulating 3HBA and gene expression levels of Foxo3, SOD1, Cata, Adiponectin, and Scd1 in adipose tissue

**Figure 2:**
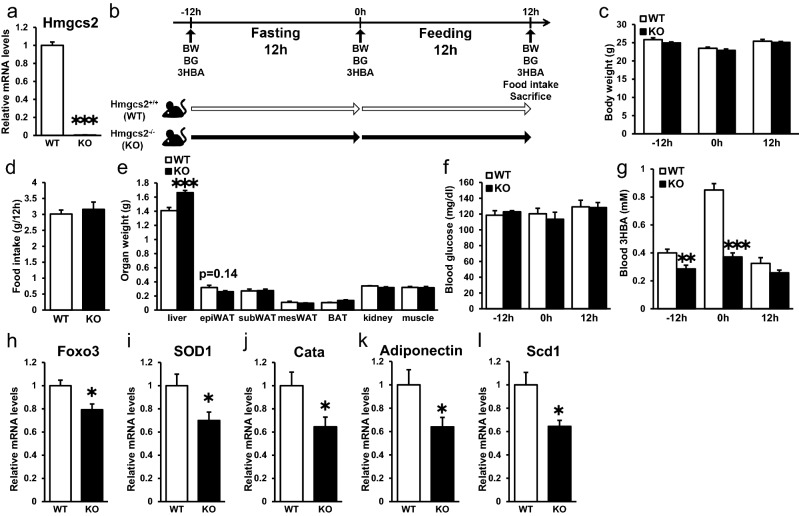
Systemic Hmgcs2 knockout mice showed decreased circulating 3HBA and gene expression levels of Foxo3, SOD1, Cata, Adiponectin, and Scd1 in adipose tissue. (**a**) qRT–PCR of Hmgcs2 in epididymal adipose tissue from Hmgcs2^+/+^ (WT) and Hmgcs2^−/−^ (KO) mice at 12 h postfeeding. WT = 8, KO = 7. (**b**) Schematic diagram of fasting and feeding subjected to WT and KO mice, including timeline for measurement of body weight, blood glucose, blood 3HBA, food intake, and sacrifice. (**c**) Body weight of WT and KO mice prefasting (− 12 h), postfasting (0 h), and postfeeding (12 h). WT = 8, KO = 7. (**d** and **e**) Food intake (**d**) and organ weight (**e**) of WT and KO mice at 12 h postfeeding. WT = 8, KO = 7. (**f** and **g**) Blood glucose (**f**) and blood 3HBA concentration (**g**) of WT and KO mice prefasting (− 12 h), postfasting (0 h), and postfeeding (12 h). WT = 8, KO = 7. (**h**–**l**) qRT–PCR of antioxidative stress factors, such as Foxo (**h**), SOD1 (**i**), and Catalase (**j**), adiponectin (**k**), and lipogenic factors, such as Scd1 (**l**), in epididymal adipose tissue from WT and KO mice at 12 h postfeeding. WT = 8, KO = 7. Data are mean ± SEM. *p < 0.05, **p < 0.01, ***p < 0.001.

To evaluate the systemic function of Hmgcs2 in vivo, we analyzed wild-type C57BL/6 male mice (Hmgcs2^+/+^; WT) and Hmgcs2 homo knockout mice (Hmgcs2^−/−^; KO). Hmgcs2 of epiWAT were deleted in KO mice (Fig. 2a). WT mice and KO mice were fasted for 12 h followed by feeding for 12 h (Fig. 2b). The body weights of prefasting, postfasting, and postfeeding mice were unaltered between groups (Fig. 2c). Food intake, weight of adipose tissues, and blood glucose levels showed no significant differences between groups (Fig. 2d–f). Recent studies have reported that antisense oligonucleotide of HMGCS2 caused insufficiency of ketogenesis resulting in accumulation of hepatic triacylglycerol^14^. In accordance with this report, liver weight of KO mice was heavier than WT mice (Fig. 2e). Plasma 3HBA concentrations at prefasting and postfasting were lower in KO mice than in WT mice (Fig. 2g). We previously reported that oxidative stress in adipose tissue is associated with adipose dysfunction^15^, and reduced oxidative stress by overexpression of antioxidative stress enzymes in adipose tissues resulted in adipose healthy expansion with enhanced lipogenesis^16^. We previously reported that 3HBA levels and mRNA expression of lipogenic factors were elevated in epiWAT of KKAy, a mouse model of obesity and type 2 diabetes, by dapagliflozin treatment^12^. Therefore, we measured oxidative stress-related gene expression in epiWAT. EpiWAT of the KO mice exhibited decreased transcript abundance of antioxidative stress factors, such as Foxo3 (Fig. 2h), SOD1 (Fig. 2i), and catalase (Fig. 2j), and adiponectin (Fig. 2k), a key adipocytokine in obesity-related disorders, which has antidiabetic, antiatherogenic, and anti-inflammatory properties^17^, compared with WT mice. Moreover, the expression levels of Scd1, a lipogenic gene, were decreased in epiWAT from KO mice compared to WT mice (Fig. 2l). Collectively, these data demonstrate that systemic Hmgcs2 knockout in mice lowered circulating 3HBA levels accompanied by decreased gene expression levels of Foxo3, SOD1, catalase, adiponectin, and Scd1 in adipose tissue.

### 3HBA enhances the gene expression levels of antioxidative stress factors, PPARγ, and lipogenic factors in epiWAT in vivo

**Figure 3:**
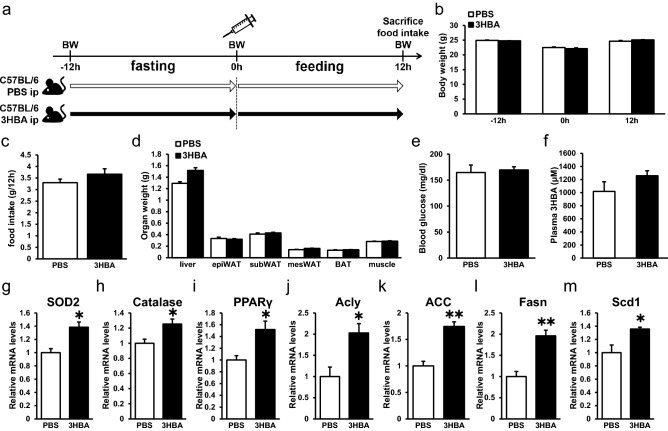
3HBA enhances the gene expression levels of antioxidative stress factors, PPARγ, and lipogenic factors in vivo. (**a**) Schematic diagram of injection of 3HBA into C57BL/6 J male mice, including timeline for measurement of body weight and food intake, intraperitoneal injection of PBS or 3HBA, and sacrifice. (**b**) Body weight of PBS- and 3HBA-injected mice prefasting (− 12 h), postfasting (0 h), and postfeeding (12 h). n = 3. (**c**–**f**) Food intake (**c**), organ weight (**d**), blood glucose (**e**), and plasma 3HBA concentration (**f**) of PBS- and 3HBA-injected mice at 12 h postfeeding. n = 3. (**g**–**m**) qRT–PCR of antioxidative stress factors, such as SOD2 (**g**) and Catalase (**h**), PPARγ (**i**), and lipogenic factors, such as Acly (**j**), ACC (**k**), Fasn (**l**), and Scd1 (**m**), in epididymal adipose tissue from PBS- and 3HBA-injected mice at 12 h postfeeding. n = 3. Data are mean ± SEM. *p < 0.05, **p < 0.01.

From these results (Fig. 2), we presumed that 3HBA might enhance mRNA expression levels of antioxidative- and de novo lipogenic enzymes in adipose tissues. To examine this hypothesis, wild-type C57BL/6 male mice were fasted for 12 h and then injected intraperitoneally with phosphate buffered saline (PBS) or 3HBA (20 mmol/kg of body weight) followed by feeding for 12 h (Fig. 3a). The body weights of prefasting, postfasting, and postfeeding mice were unaltered between PBS- and 3HBA-injected mice (Fig. 3b). Food intake, organ weight, and blood glucose showed no significant differences between groups (Fig. 3c–e). Plasma 3HBA concentrations showed an increasing trend in 3HBA-injected mice compared to PBS-injected mice (Fig. 3f; *p* = 0.2251). EpiWAT of the 3HBA-injected mice exhibited increased transcript abundance of antioxidative stress factors, such as SOD2 (Fig. 3g) and catalase (Fig. 3h), and PPARγ (Fig. 3i), a critical factor regulating lipid metabolism, adipocytokine secretion, and insulin sensitivity in mature adipocytes^18^, compared with PBS. Moreover, the expression levels of lipogenic genes, such as Acly (Fig. 3j), ACC (Fig. 3k), Fasn (Fig. 3l) and Scd1 (Fig. 3m) were increased in epiWAT from 3HBA-injected mice compared to PBS-injected mice. Collectively, these data demonstrate that single-bolus injection of 3HBA enhanced the mRNA expression levels of antioxidative stress factors, PPARγ, and lipogenic factors in adipose tissue.

### 3HBA exerts beneficial effects on adipocytes by reducing ROS via augmentation of antioxidative stress factors and inducing PPARγ, adiponectin, insulin signaling, and lipogenic factors in vitro

**Figure 4:**
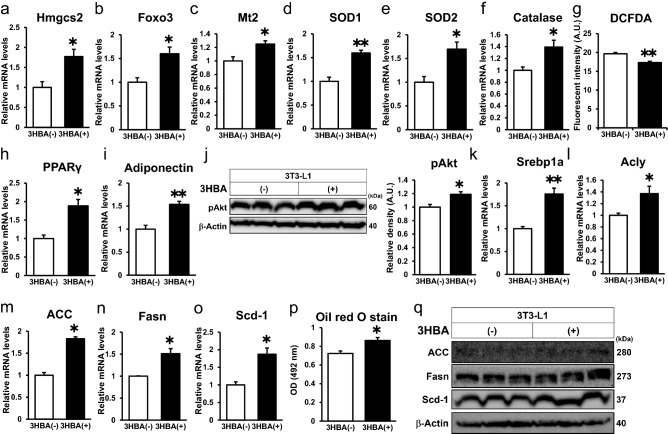
3HBA exerts beneficial effects on adipocytes by reducing ROS levels via augmentation of antioxidative stress factors and inducing PPARγ, insulin signaling, and lipogenic factors in vitro. On day 7 after 3T3-L1 adipocytes were differentiated, the 3T3-L1 adipocytes were maintained in serum-free DMEM composed of 2.5 mM glucose and 0 mM or 10 mM 3HBA for 24 h. On day 8 after differentiation, the 3T3-L1 adipocytes were additively stimulated with 1 nM insulin for 24 h, followed by harvesting on day 9 after differentiation. (**a**–**f**) qRT–PCR of Hmgcs2 and antioxidative stress factors. n = 3. (**g**) Cellular ROS detected by 2’,7’-dichlorofluorescein diacetate (DCFDA) assay. n = 3. (**h** and **i**) qRT–PCR of PPARγ (**h**) and adiponectin (**i**). n = 3. (**j**) Western blot of pAkt and β-Actin. *Left panel;* Representative western blot analysis. *Right panel;* Quantitative analysis of pAkt in the left panel. n = 3. (**k**–**o**) qRT–PCR of lipogenic factors. n = 3. (**p**) Oil red O stain (OD = 492 nm). n = 3. (**q**) Western blot of lipogenic factors and β-Actin. n = 3. Here cropped blots were displayed and all full-length blots are included in the Supplementary Figure S4. Data are mean ± SEM. *p < 0.05, **p < 0.01. A.U., Arbitrary Unit.

In adipose tissues, the expression of lipogenic genes increases under refeeding conditions compared with fasting conditions^19^. In our mouse model, treatment with 3HBA induced an abundance of antioxidative stress factors, PPARγ, and lipogenic factors in adipose tissues under feeding conditions (Fig. 3). To further investigate the direct effect of 3HBA on adipocytes, we incubated 3T3-L1 adipocytes with or without 10 mM 3-HBA at a similar level in the plasma of fasting mice (Fig. 1d) in the presence of insulin to mimic feeding conditions. Treatment with 3HBA increased the gene expression of Hmgcs2 (Fig. 4a) and antioxidative stress factors, such as Foxo3, Mt2, SOD1, SOD2, and catalase, in 3T3-L1 adipocytes (Fig. 4b–f). Foxo3 is a transcription factor that induces cell cycle arrest and resistance to oxidative stress^20^. Mt2, SOD1, SOD2, and catalase protect cells against oxidative stress^16,21^. DCFDA is a cell-permeable fluorescent indicator for reactive oxygen species in living cells. In parallel with the upregulation of antioxidative stress factors, the DCFDA assay indicated that treatment with 3HBA reduced reactive oxygen species (ROS) production in 3T3-L1 adipocytes (Fig. 4g). PPARγ is a critical transcription factor that maintains adipocyte function, such as insulin signaling and lipogenesis^22^, and we previously reported that ROS scavengers increased the expression of PPARγ and its downstream factor adiponectin and improved adipocyte function^15^. In accordance with these reports, treatment with 3HBA increased the mRNA expression of PPARγ and adiponectin (Fig. 4h and i) and enhanced insulin-induced phosphorylation of Akt at serine 473 (pAkt) (Fig. 4j). Treatment with 3HBA significantly enhanced adiponectin secretion into the culture medium of 3T3-L1 adipocytes compared with those without 3HBA (Supplementary Figure S1). Furthermore, treatment with 3HBA increased the mRNA expression of lipogenic genes, such as Srebp1a, Acly, ACC, Fasn and Scd1, in 3T3-L1 adipocytes (Fig. 4k–o). 3HBA significantly enhanced insulin-induced lipid accumulation measured by Oil red O stain in 3T3-L1 adipocytes (Fig. 4p). In this condition, 3HBA also induced glycerol secretion into the culture medium of 3T3-L1 adipocytes compared with those without 3HBA (Supplementary Figure S2), suggesting that lipogenic activity is induced more than lipolytic activity resulting in lipid accumulation by 3HBA treatment. However, treatment with 3HBA showed no change in the abundance of key lipogenic proteins, such as ACC, Fasn or Scd1, in 3T3-L1 adipocytes (Fig. 4q). Together, these data demonstrate that 3HBA, at physiological concentrations, exerts beneficial effects on adipocytes, accompanied by reduced ROS levels, via augmentation of antioxidative stress factors and induction of PPARγ, adiponectin, insulin signaling, and lipogenic factors in vitro.

### Overexpression of Hmgcs2 is sufficient to induce 3HBA production, antioxidative stress factors, PPARγ, and lipogenic factors in adipocytes

**Figure 5:**
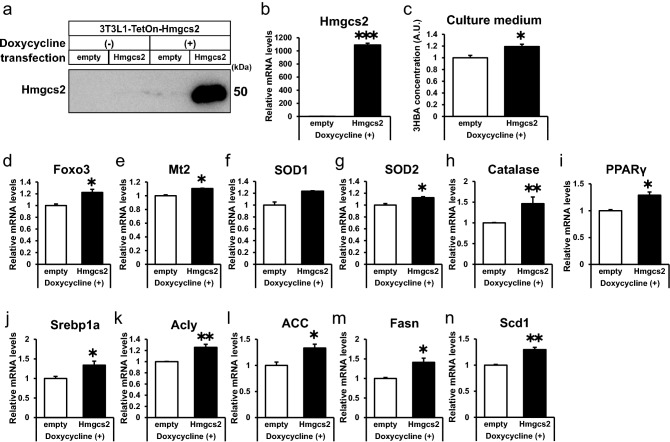
Overexpression of Hmgcs2 is sufficient to induce 3HBA production, antioxidative stress factors, PPARγ, and lipogenic factors in adipocytes. On day 5 after 3T3-L1-TetON-empty and 3T3-L1-TetON-Hmgcs2 adipocytes were differentiated, the adipocytes were treated with 2 µg/mL doxycycline for 48 h. On day 7 after differentiation, these adipocytes were maintained in serum-free DMEM composed of 25 mM glucose and 1 nM insulin for 24 h, followed by harvesting on day 8 after differentiation. (**a**) Western blot of Hmgcs2. n = 1. (**b**) qRT–PCR of Hmgcs2. n = 3. (**c**) 3HBA concentration in culture supernate. n = 3. (**d**–**h**) qRT–PCR of antioxidative stress factors. n = 3. (**i**) qRT–PCR of PPARγ. n = 3. (**j**–**n**) qRT–PCR of lipogenic factors. n = 3. Data are mean ± SEM. *p < 0.05, **p < 0.01, ***p < 0.001. A.U., Arbitrary Unit.

To further confirm the significance of Hmgcs2 in adipocytes, we introduced 3T3-L1 cells with stable expression of pRetroX-Tet-On with pRetroX-Tight-Hmgcs2 or pRetroX-Tight-empty and thus established 3T3-L1-TetON- Hmgcs2 (Hmgcs2-L1) and 3T3-L1-TetON-empty cells (empty-L1). Hmgcs2-L1 cells conditionally expressed ectopic protein and mRNA of Hmgcs2 when treated with doxycycline followed by insulin treatment (Fig. 5a and b). As expected, treatment with doxycycline elevated 3HBA secretion into the culture medium of Hmgcs2-L1 adipocytes compared with empty-L1 adipocytes (Fig. 5c). There was more gene expression of antioxidative stress factors (Foxo3, Mt2, SOD2, and catalase) (Fig. 5d-5h), PPARγ (Fig. 5i), and lipogenic factors (Srebp1a, Acly, ACC, Fasn, and Scd1) (Fig. 5j–n) in Hmgcs2-L1 adipocytes than in empty-L1 adipocytes. Altogether, these data demonstrate that overexpression of Hmgcs2 is sufficient to induce 3HBA production, antioxidative stress factors, PPARγ, and lipogenic factors in adipocytes.

### Adipocytes express Hmgcs2 and produce and secrete 3HBA

**Figure 6:**
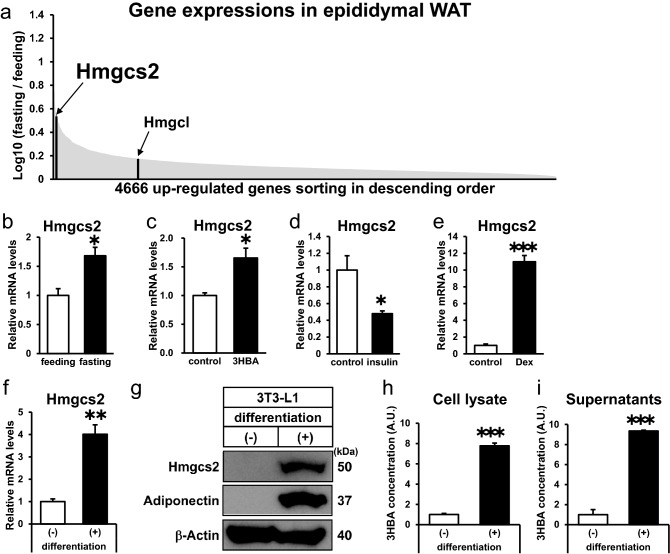
Hmgcs2 is expressed in adipose tissue in vivo and in vitro, and adipocytes produce and secrete 3HBA. (**a**) Schematic diagram of microarray analysis to identify fasting-inducing genes expressed in adipose tissue. The genes upregulated by fasting are sorted in descending order. The following Gene Expression Omnibus DataSet was used for the analysis: GSE46495 (fold-change > 2.0, *p* < 0.05; 4666 genes). (**b**) qRT–PCR of Hmgcs2 in epididymal adipose tissue from C57BL/6 J mice after 12 h of feeding and fasting. n = 3. (**c**–**e**) qRT–PCR of Hmgcs2 in differentiaed 3T3-L1 adipocytes after 24 h of treatment with 10 mM 3HBA (**c**), 1 nM insulin (**d**), and 1 µM dexamethasone (**e**). n = 3. (**f**) qRT–PCR of Hmgcs2. n = 3. (**g**) Intracellular protein of Hmgcs2. n = 1. (**h** and **i**) 3HBA concentrations in cell lysate (**h**) and cell culture supernatant (**i**) of differentiated and undifferentiated 3T3-L1 adipocytes. n = 3. For measurement of 3HBA, cell lysate was normalized per well of a 6 well plate, and culture supernatant was normalized per 2 mL media for a well of 6 well plate. Here cropped blots were displayed and all full-length blots are included in the supplemental Fig. S2. Data are mean ± SEM. *p < 0.05, **p < 0.01, ***p < 0.001. A.U., Arbitrary Unit.

To reveal the physiological regulation of ketogenesis-associated genes in adipose tissues (Fig. 1a), we compiled microarray datasets of upregulated genes by fasting in epididymal adipose tissue (epiWAT) of mice (GSE46495)^23^. In this dataset, the expression levels of Hmgcs2 and Hmgcl were significantly higher in epiWAT of fasting mice than in feeding mice. Sorting 4,666 upregulated genes in descending order, Hmgcs2 was among the most highly ranked genes (Fig. 6a). In epiWAT of fasting mice, mRNA expression of Hmgcs2 was elevated in comparison to feeding mice (Fig. 6b), consistent with analysis of microarray datasets (Fig. 6a).

Next, to reveal the regulation of the Hmgcs2 gene in vitro, we incubated 3T3-L1 adipocytes with 3-HBA to mimic fasting conditions in vitro. Hmgcs2 transcript levels were strengthened by 3HBA treatment (Fig. 6c). To further reveal the hormonal regulation of Hmgcs2, 3T3-L1 adipocytes were treated with insulin or dexamethasone. Gene expression of Hmgcs2 was downregulated by insulin and markedly upregulated by dexamethasone (Fig. 6d and e). Considering that plasma insulin levels were decreased whereas plasma 3-HBA and dexamethasone levels were increased under fasting conditions, the gene expression of Hmgcs2 is reasonably regulated by these factors in adipocytes.

Moreover, we compared the expression of Hmgcs2 between 3T3-L1 preadipocytes and adipocytes. Gene and protein expression of Hmgcs2 was augmented in differentiated 3T3-L1 adipocytes relative to undifferentiated 3T3-L1 adipocytes (Fig. 6f and g). Strikingly, we observed a marked elevation of 3HBA concentrations in both cell lysate and cell culture supernatants in differentiated 3T3-L1 adipocytes compared to undifferentiated 3T3-L1 adipocytes (Fig. 6h and i), suggesting that 3HBA was endogenously produced and secreted from differentiated adipocytes. Together, these data demonstrate that Hmgcs2 is expressed in adipose tissues and adipocytes and that adipocytes produce and secrete 3HBA.

### Hmgcs2 regulates antioxidative stress factors and ROS, PPARγ, adiponectin, and lipogenic factors in an autocrine manner by endogenous 3HBA production in adipocytes

**Figure 7:**
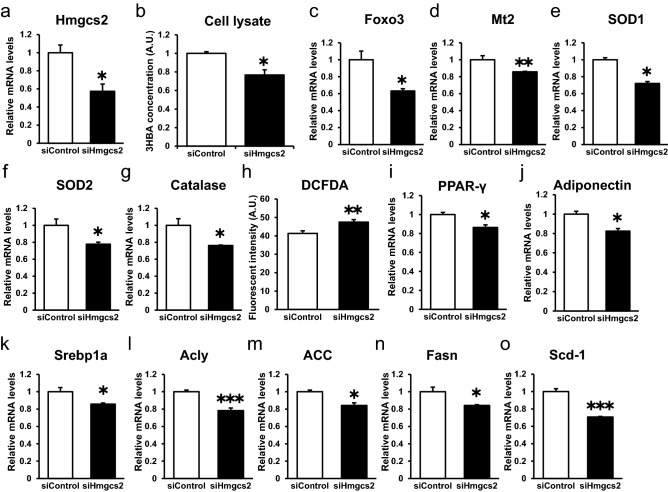
Hmgcs2 regulates antioxidative stress factors and ROS, PPARγ, and lipogenic factors in an autocrine manner by endogenous 3HBA production in adipocytes. On day 3 after 3T3-L1 adipocytes were differentiated, siRNA was introduced by reverse transfection for 48 h, and forward transfection was performed for 48 h. On day 7 after differentiation, the 3T3-L1 adipocytes were maintained in serum-free DMEM composed of 25 mM glucose and 1 nM insulin for 24 h, followed by harvest on day 8 after differentiation. (**a**) qRT–PCR of Hmgcs2. (**b**) 3HBA concentration in cell lysate. n = 3. (**c**–**g**) qRT–PCR of antioxidative stress factors. n = 3. (**h**) Cellular ROS detected by 2′,7′-dichlorofluorescein diacetate (DCFDA) assay. n = 3. (**i**,**j**) qRT–PCR of PPARγ and Adiponectin. n = 3. (**k**–**o**) qRT–PCR of lipogenic factors. n = 3. Data are mean ± SEM. *p < 0.05, **p < 0.01, ***p < 0.001. A.U., Arbitrary Unit.

As shown in Fig. 6, we confirmed the gene and protein expression of Hmgcs2 and endogenous production of 3HBA in 3T3-L1 adipocytes. Furthermore, 3HBA treatment increased Hmgcs2 mRNA abundance in adipocytes (Fig. 4a). Based on these findings, we hypothesized that adipocyte-derived 3HBA acts in an autocrine manner to control antioxidative stress factors, PPARγ and lipogenesis factors in adipocytes. To confirm this hypothesis, we estimated the autocrine action of Hmgcs2 in 3T3-L1 adipocytes. We knocked down Hmgcs2 by siRNA followed by insulin treatment. The gene expression of Hmgcs2 was reduced by siRNA against Hmgcs2 (Fig. 7a). Under this condition, intracellular 3HBA levels were reduced (Fig. 7b). Additionally, knockdown of Hmgcs2 decreased the gene expression of antioxidative stress factors such as Foxo3, Mt2, SOD1, SOD2, and catalase (Fig. 7c–g) and increased ROS production, as detected by DCFDA assay, in 3T3-L1 adipocytes (Fig. 7h). Furthermore, knockdown of Hmgcs2 reduced the gene expression levels of PPARγ, adiponectin (Fig. 7i and j) and lipogenic factors such as Srebp1a, Acly, ACC, Fasn, and Scd1 (Fig. 7k–o) in 3T3-L1 adipocytes. Taken together, these data demonstrate that Hmgcs2 regulates antioxidative stress factors, ROS, PPARγ, adiponectin, and lipogenic factors in an autocrine manner by endogenous 3HBA production in adipocytes.

## Discussion

**Figure 8:**
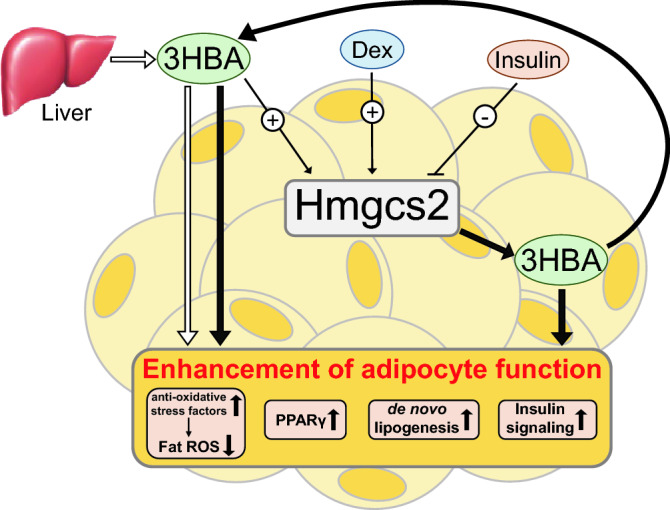
A working hypothesis illustrating how 3HBA contributes to the enhanced function of adipocytes. Circulating 3HBA is primarily synthesized in the liver and secreted. In adipocytes, Hmgcs2 is upregulated by 3HBA and dexamethasone (Dex) but downregulated by insulin. 3HBA enhances the function of adipocytes by reducing fat ROS by inducing antioxidative stress factors and intensifying PPARγ, de novo lipogenesis, and insulin signaling in an endocrine (thick white arrow) and autocrine/paracrine manner through endogenous Hmgcs2 expression followed by the synthesis of 3HBA in adipocytes (thick black arrow).

In the current study, we found that adipose tissue and adipocytes express Hmgcs2 which is upregulated by 3HBA and dexamethasone, and downregulated by insulin, and that adipocytes produce and secrete 3HBA. Moreover, 3HBA plays significant roles in adipocytes to enhance the gene expression levels of antioxidative stress factors, PPARγ, and lipogenic factors with reduced ROS levels, probably in an endocrine and autocrine/paracrine manner (Fig. 8). In addition, Hmgcs2 gene expression was increased by fasting in adipose tissues of mice, suggesting the physiological role of endogenous ketogenesis of adipocytes in vivo.

Previously, we reported that oxidative stress in adipose tissue suppresses the gene expression of PPARγ^15^. In addition, we established adipose ROS-eliminated and adipose ROS-augmented mice through the genetic manipulation of antioxidant-related genes. Adipose ROS-eliminated mice exhibited white adipose expansion with elevated lipogenic genes and improved insulin signaling. Conversely, adipose ROS-augmented mice exhibited restricted adipose expansion with reduced lipogenic genes and accelerated insulin resistance, indicating the significant role of ROS in regulating lipogenic genes and insulin signaling in adipose tissues^16^. On the other hand, it was reported that treatment with 3HBA epigenetically induced antioxidative enzymes in the kidney through HDAC inhibition, resulting in reduced ROS^9^. In the current study, we indicated that 3HBA reduced ROS, increased expression of PPARγ and lipogenic genes, and enhanced insulin signaling in adipocytes. One possible explanation is that the effect of 3HBA on enhancing adipocyte function might be attributed to reduced ROS levels through induction of antioxidative enzymes by epigenetic regulation.

Ketone bodies, including 3HBA, are not only an alternative energy source under starvation but also play multiple roles as signaling molecules^1^. For instance, 3HBA induces histone b-hydroxybutyrylation, a recently described epigenetic mark that is involved in metabolic regulation in the liver^11^, and augments the expression of adiponectin in adipocytes^12^. In the intestine, a ketogenic diet increased serum and intestinal 3HBA levels to robustly oscillate in a circadian manner. This oscillation of 3HBA was coupled to tissue-specific cyclic histone deacetylase (HDAC) activity and histone acetylation, resulting in nuclear accumulation of PPARα, a metabolic master regulator, in the intestine^24^. On the other hand, 3HBA acts as a ligand of the G protein-coupled receptor (GPR). 3HBA is a ligand of GPR41 and regulates sympathetic nervous system activity and energy expenditure^25^. 3HBA is also a ligand of GPR109A and suppresses lipolysis through activation of immune cells^26,27^. However, gene expressions of both GPR41 and GPR109A in epiWAT of mice were not detected (GSE46495)^23^. Thus, ketone bodies, including 3HBA, exert various effects through multiple molecular mechanisms. Nevertheless, few studies have been reported on the relationship between 3HBA and adipocytes. Further studies are required to determine the mechanism by which 3HBA enhances the physiological function of adipocytes.

During fasting, circulating insulin levels are low, and the primary energy source shifts from glucose to free fatty acids (FFAs) released from adipocytes by lipolysis. FFAs are converted to ketone bodies mainly in the liver and transported to extrahepatic tissues. Therefore, prolonged fasting is accompanied by low serum insulin levels, elevated serum ketone bodies, and reduced storage of triglycerides in adipocytes. In the initial phase of feeding following prolonged fasting, plasma insulin levels rise quickly, while plasma 3HBA levels fall slowly and remain high in human subjects^28^. In the current study, 3HBA did not induce PPARγ or lipogenic genes in the absence of insulin (Supplementary Figure S3) but increased them accompanied by lipid accumulation in the presence of insulin (Figs. 3 and 4). In addition, 3HBA intensified insulin signaling (Fig. 4). In the current study, 3HBA induced lipid accumulation without changes in lipogenic protein expressions (Fig. 4). Insulin activates and ROS inhibits lipogenic activity^29,30^. In addition, insulin increases glucose uptake, and glucose is a substrate for lipogenesis. Moreover, 3HBA itself is a substrate for lipogenesis. Taken together, 3HBA potentiates lipid accumulation possibly by these multiple mechanisms to restore excessively mobilized and exhausted adipose tissue after prolonged fasting.

In conclusion, our results revealed for the first time that adipocytes express Hmgcs2 and produce 3HBA and that 3HBA enhances adipocyte function in endocrine and autocrine/paracrine manners. Further investigations are required to reveal the physiological and pathological significance of ketogenesis in adipocytes in vivo by establishing an adipocyte-specific Hmgcs2-deficient mouse model.

## Methods

### Animal studies and approval

Seven-week-old male C57BL/6 J mice were obtained from CLEA Japan (Tokyo, Japan) and acclimated for more than 1 week before the experiment. Mice were housed individually in sterile cages, maintained in a room under controlled temperature (23 ± 1.5 °C) and humidity (45 ± 15%) on a 12-h dark/12-h light cycle, and had free access to water and normal chow diets (MF; Oriental Yeast, Tokyo). Samples from six tissues were collected, including epididymal fat, subcutaneous fat, mesenteric fat, BAT, liver, and skeletal muscle (gastrocnemius muscle). The experimental protocol was approved by the Ethics Review Committee for Animal Experimentation of Osaka University, Graduate School of Medicine. These mouse studies were approved by the Ethics Review Committee for Animal Experimentation of Osaka University, Graduate School of Medicine, and carried out in accordance with the Institutional Animal Care and Use Committee Guidelines of Osaka University.

WT (Hmgcs2^+/+^) mice and KO (Hmgcs2^−/−^) mice were previously established by Hiroshi Maegawa’s lab^31^. Briefly, a mouse model harboring a deletion mutation in exon 2 of the mouse Hmgcs2 gene was generated using the CRISPR/Cas9 system. The following sequences were used for single guide RNA (sgRNA) synthesis: sgRNA1, TGGAACGCACAAAGCTGCCG; sgRNA2, GTGCCTGCAGTGGTACAGA. Cas9 mRNA and sgRNAs (Cas9 mRNA: gRNA1gg:gRNA2cc = 2:1:1) were microinjected into fertilized C57BL/6 J mouse embryos. Among these, one mouse line with a 38-bp deletion mutation in exon 2, which led to a frameshift and subsequent complete deletion of Hmgcs2 protein synthesis, was selected. Homozygous Hmgcs2^−/−^ mice were born from heterozygous intercrosses and used for phenotypic analyses in parallel with age- and sex-matched wild-type littermates as a control group. These mice were housed in a temperature-controlled environment (23 °C) with a 12-h light and 12-h dark (20:00–08:00) photoperiod and cared for in facilities operated by the Research Center for Animal Life Science at Shiga University of Medical Science. CLEA Rodent Diet (#CE-2, CLEA Japan) was used as the standard chow in the center. The experimental protocol was approved by the Gene Recombination Experiment Safety Committee and Research Center for Animal Life Science (RCALS) at Shiga University of Medical Science.

In the feeding versus fasting experiment, 10-week-old male C57BL/6 J mice were subjected to feeding or fasting conditions for 12 h and then anesthetized with isoflurane, and blood samples were collected from the heart, followed by sacrifice. Body weight was measured at the start and end of the experiment.

In the WT (Hmgcs2 + / +) mice versus KO (Hmgcs2 −/−) mice experiment, 12- or 13-week-old male mice were subjected to fasting condition for 12 h, followed by feeding for 12 h, and then anesthetized with isoflurane and blood samples were collected from the heart, followed by sacrifice. Body weight, blood glucose, and blood 3HBA were measured at prefasting, postfasting, and postfeeding, and food intake was determined by weighing the metal cage top, including the food at the end of the experiment.

In the injection of PBS or 3HBA experiment, 8-week-old male C57BL/6 J mice were subjected to fasting conditions for 12 h before being injected intraperitoneally with PBS or 3HBA (20 mmol/kg of body weight), fed for 12 h, and then anesthetized with isoflurane. Blood samples were collected from the heart, followed by sacrifice. Body weight was measured at prefasting, postfasting, and postfeeding, and food intake was determined by weighing the metal cage top, including the food at the end of the experiment.

### Measurements of blood parameters

Blood glucose levels were measured by tail vein sampling before anesthesia using the Glutest Neo alpha (Sanwa Kagaku Kenkyusho, Nagoya, Japan). Blood 3HBA levels were measured by tail vein sampling before anesthesia using the PRECISION XCEED (Ketometer) (Abbott, Tokyo, Japan). Plasma concentrations of 3HBA were measured using a beta Hydroxybutyrate (beta HB) Assay Kit (Abcam) according to the instructions provided by the manufacturer.

### Cell cultures and differentiation

3T3-L1 adipocytes were cultured in DMEM (Nacalai Tesque, Kyoto, Japan) supplemented with 10% FBS until two days post-confluence. Then (day 0), the cells were treated with or without adipogenic mixture containing 0.5 mM 3-isobutyl-1-methylxanthine (Nacalai), 1 μM dexamethasone (Sigma-Aldrich, St. Louis, MO), and 1 μM insulin (Nacalai) for 48 h. After 48 h (day 2), medium was replaced with DMEM supplemented with 10% FBS. Until day 7, the cells were maintained in DMEM with 10% FBS.

### Measurement of 3HBA abundance in cell lysate and culture supernatant

3-HBA abundance in cell lysate and culture supernatant was measured using a beta Hydroxybutyrate (beta HB) Assay Kit (Abcam) according to the instructions provided by the manufacturer.

### Measurement of intracellular ROS

Intracellular ROS were measured using the DCFDA Cellular ROS Detection Assay Kit (Abcam) with the instructions provided by the manufacturer. Briefly, on day 9 of treatment of 3HBA on 3T3-L1 adipocytes (Fig. 4), or on day 8 of transfection with small interfering RNA (Fig. 7), culture medium was replaced with Krebs–Ringer Bicarbonate buffer (KRBB), composed of 25 mM NaHCO_3_ (Nacalai), 119 mM NaCl (Nacalai), 4.74 mM KCl (Nacalai), 1.19 mM MgCl_2_ (Nacalai), 1.19 mM KH_2_PO_4_ (Nacalai), 2.54 mM CaCl_2_ (Nacalai), 10 mM 4-(2-hydroxyethyl)-1-iperazineethanesulfonic acid (Nacalai), and 0.05 mM Bovine Serum Albumin solution (Sigma), supplemented with 10 μM DCFDA. After staining with DCFDA for 1 h at 37 ℃ under 5% CO_2_, 3T3-L1 adipocytes were wash 3 times with PBS and detected by fluorescence spectroscopy with excitation / emission at 485 nm / 535 nm.

### Effects of 3HBA on 3T3-L1 adipocytes

On day 7 after differentiation, the medium of 3T3-L1 adipocytes was replaced with DMEM composed of 2.5 mM glucose with 10 mM concentrations of 3-hydroxybutyric acid (Sigma–Aldrich). After 24 h (day 8), 3T3-L1 adipocytes were additively stimulated by 1 nM insulin for 24 h, followed by harvesting (day 9).

### Effects of insulin and dexamethasone on 3T3-L1 adipocytes

On day 7 after differentiation, the medium of 3T3-L1 adipocytes was replaced with DMEM composed of 25 mM glucose with 1 nM insulin or 1 µM dexamethasone for 24 h, followed by harvesting (day 8).

### Transfection with small interfering RNA

3T3-L1 adipocytes were harvested, and small interfering (si)RNA was introduced by reverse transfection on day 3 after differentiation, followed by forward transfection on day 5, using the Lipofectamine RNAiMAX Reagent (Invitrogen) according to the instructions provided by the manufacturer. On day 7 after differentiation, the 3T3-L1 adipocytes were maintained in serum-free DMEM composed of 25 mM glucose and 1 nM insulin for 24 h, followed by harvest (day 8).

### Retroviral infection

Platinum-E cells were transfected with pRetroX-Tet-On Advanced (TaKaRa), and RetroX-Tight-Pur (TaKaRa) harboring the mouse Hmgcs2 gene. The media containing the retroviruses were harvested 48 h after transfection, filtered, and transferred to 3T3-L1 cells. Infected cells were selected with 200 mg/mL G418, 400 mg/mL hygromycin, and 1 mg/mL puromycin.

### Doxycycline-inducible overexpression of Hmgcs2 on 3T3-L1 adipocytes

On day 5 after 3T3-L1-TetON-empty and 3T3-L1-TetON-Hmgcs2 adipocytes were differentiated, the adipocytes were treated with 2 µg/mL doxycycline for 48 h. On day 7 after differentiation, these adipocytes were maintained in serum-free DMEM composed of 25 mM glucose and 1 nM insulin for 24 h, followed by harvesting on day 8 after differentiation.

### RNA extraction, cDNA synthesis, and quantitative real-time PCR

Total RNA was isolated with TRI-Reagent (Sigma–Aldrich) based on the method recommended by the manufacturer. cDNA was synthesized using Transcriptor Universal cDNA Master Mix (Roche) and was subjected to RT-PCR using FastStart Essential DNA Green Master Mix (Roche) on a LightCycler® 96 Instrument (Roche) according to the protocol provided by the manufacturer. The primers used are described in Supplementary Table 1.

### Western blot analysis

Cell lysates of 3T3-L1 cells were collected. Protein levels were determined by blotting with phospho-Akt (S4060, Ser473) were purchased from Cell Signaling Tschnologies. β-actin (A5441) were purchased from Sigma–Aldrich. Hmgcs2 (ab137043) were purchased from Abcam. Chemiluminescent images were obtained by ChemiDoc Touch imaging system (Biorad, Hercules, CA) with Optimal Auto-exposure mode.

### Statistical analysis

All data were expressed as the mean ± SEM values. Differences between two groups were examined for statistical significance by Student’s t-test. A *P* value < 0.05 denoted the presence of a statistically significant difference.

## Supplementary Information

Supplementary Information.

## Data Availability

All data generated or analyzed during this study are included in this published article and its supplementary information files.

## Abbreviations

ACC: Acetyl-Coenzyme A carboxylase alpha
Acly: Adenosine triphosphate citrate lyase
BAT: Brown adipose tissue
Bdh1: β-Hydroxybutyrate dehydrogenase
DCFDA: Dichlorodihydrofluorescein diacetate
epiWAT: Epididymal white adipose tissue
Fasn: Fatty acid synthase
FFAs: Free fatty acids
Foxo3: Forkhead box O3
GPR: G protein-coupled receptor
3HBA: 3-Hydroxybutyric acid
HDAC: Histone deacetylases
Hmgcl: 3-Hydroxy-3-methylglutaryl-CoA lyase
Hmgcs2: 3-Hydroxy-3-methylglutaryl-CoA synthetase 2
ISCs: Intestinal stem cells
mesWAT: Mesenteric white adipose tissue
MCT: Monocarboxylate transporter
MnSOD: Manganese superoxide dismutase
Mt2: Metallothionein 2
PPARα: Peroxisome proliferative activated receptor alpha
PPARγ: Peroxisome proliferative activated receptor gamma
ROS: Reactive oxygen species
RPE: Retinal pigment epithelial
Scd1: Stearoyl-Coenzyme A desaturase 1
SOD1: Superoxide dismutase 1
SOD2: Superoxide dismutase 2
Srebp1a: Sterol regulatory element binding transcription protein 1
subWAT: Subcutaneous white adipose tissue
