# Supplementary material for: Ketone body 3-hydroxybutyrate enhances adipocyte function
Source: Sci Rep. 2022 Jun 16;12:10080. doi: 10.1038/s41598-022-14268-w (PMC9203800; doi:10.1038/s41598-022-14268-w)
Supplement: Supplementary file 1 — Supplementary Information. [file 41598_2022_14268_MOESM1_ESM.docx]

**Supplemental information**


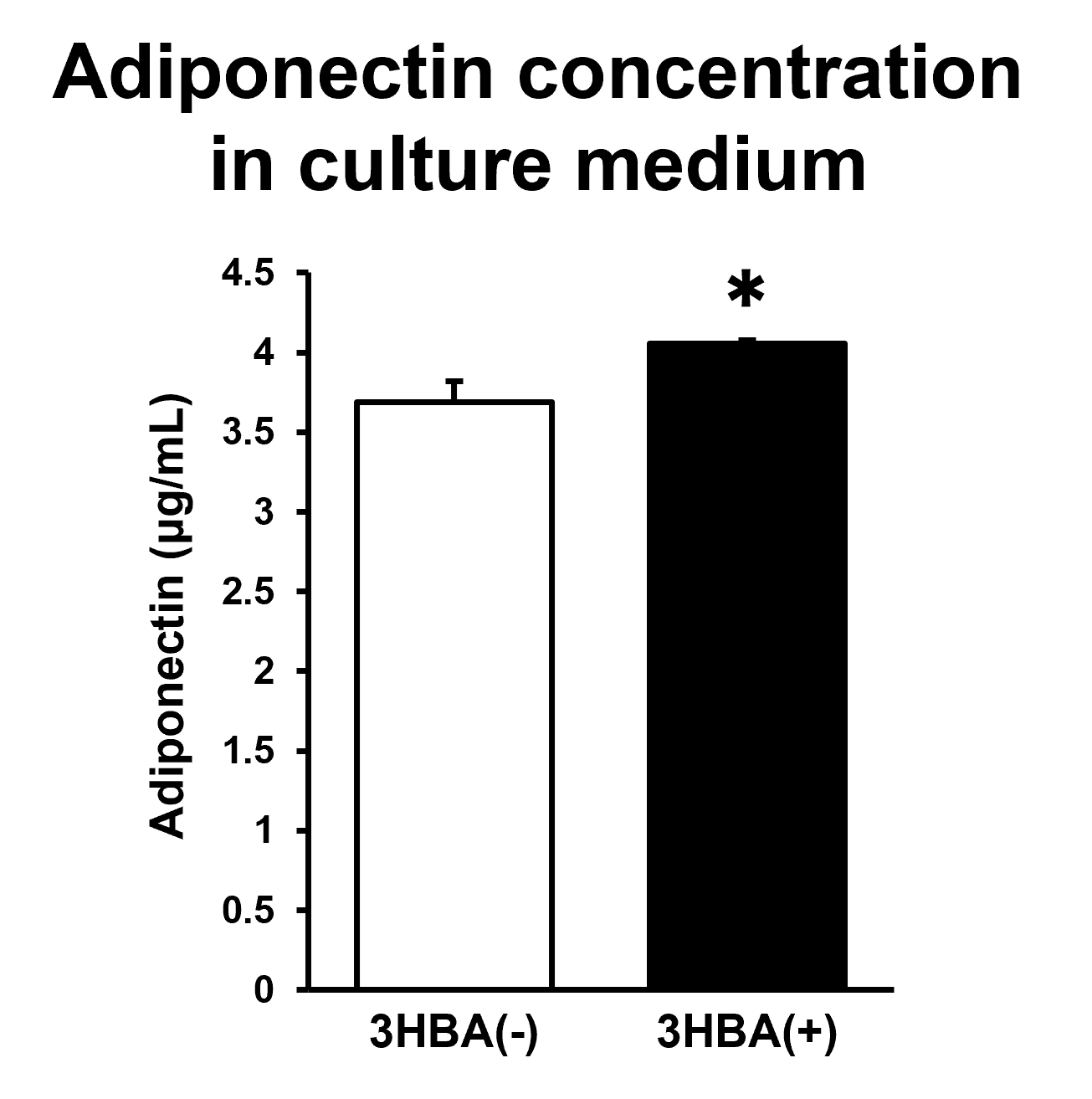


**Figure S1. 3HBA enhanced adiponectin secretion into culture medium of 3T3-L1 adipocytes.** n=3. Data are mean ± SEM. *p<0.05.


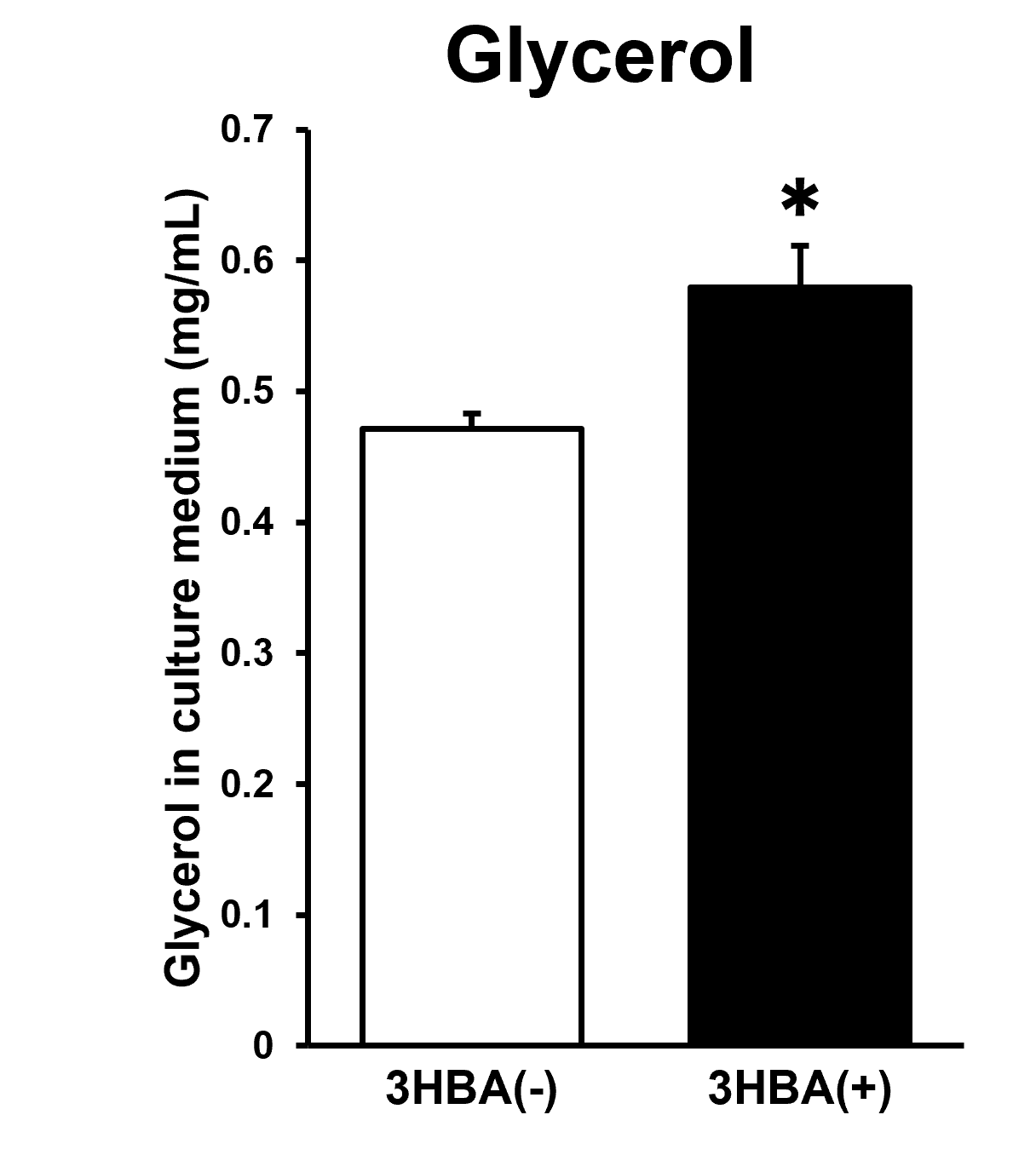


**Figure S2. 3HBA induced lipolysis of 3T3-L1 adipocytes.** Concentrations of glycerol in culture medium of 3T3-L1 adipocytes. n=3. Data are mean ± SEM. *p<0.05.


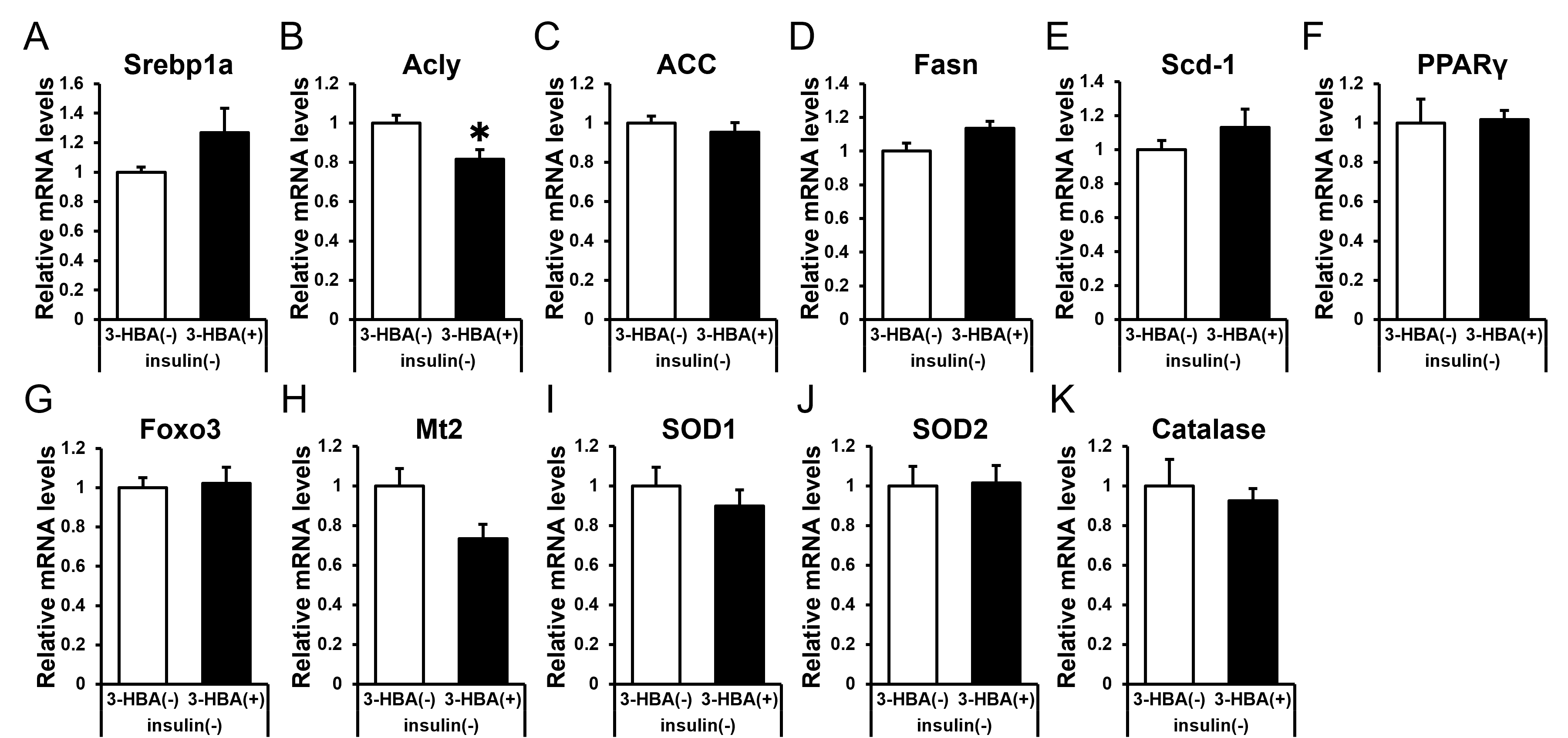


**Figure S3. 3HBA does not induce anti-oxidative stress factors, PPARγ, and lipogenic factors in the absence of insulin *in vitro*.** On day 7 after 3T3-L1 adipocytes were differentiated, the 3T3-L1 adipocytes were maintained in serum-free DMEM composed of 2.5 mM glucose and 0 mM or 10 mM 3HBA for 48 hour, followed by harvested on day 9 after differentiation. (A-E) qRT-PCR of anti-oxidative stress factors. n=3. (F) qRT-PCR of PPARγ. n=3. (G-K) qRT-PCR of lipogenic factors. n=3. Data are mean ± SEM. *p<0.05.

**A**


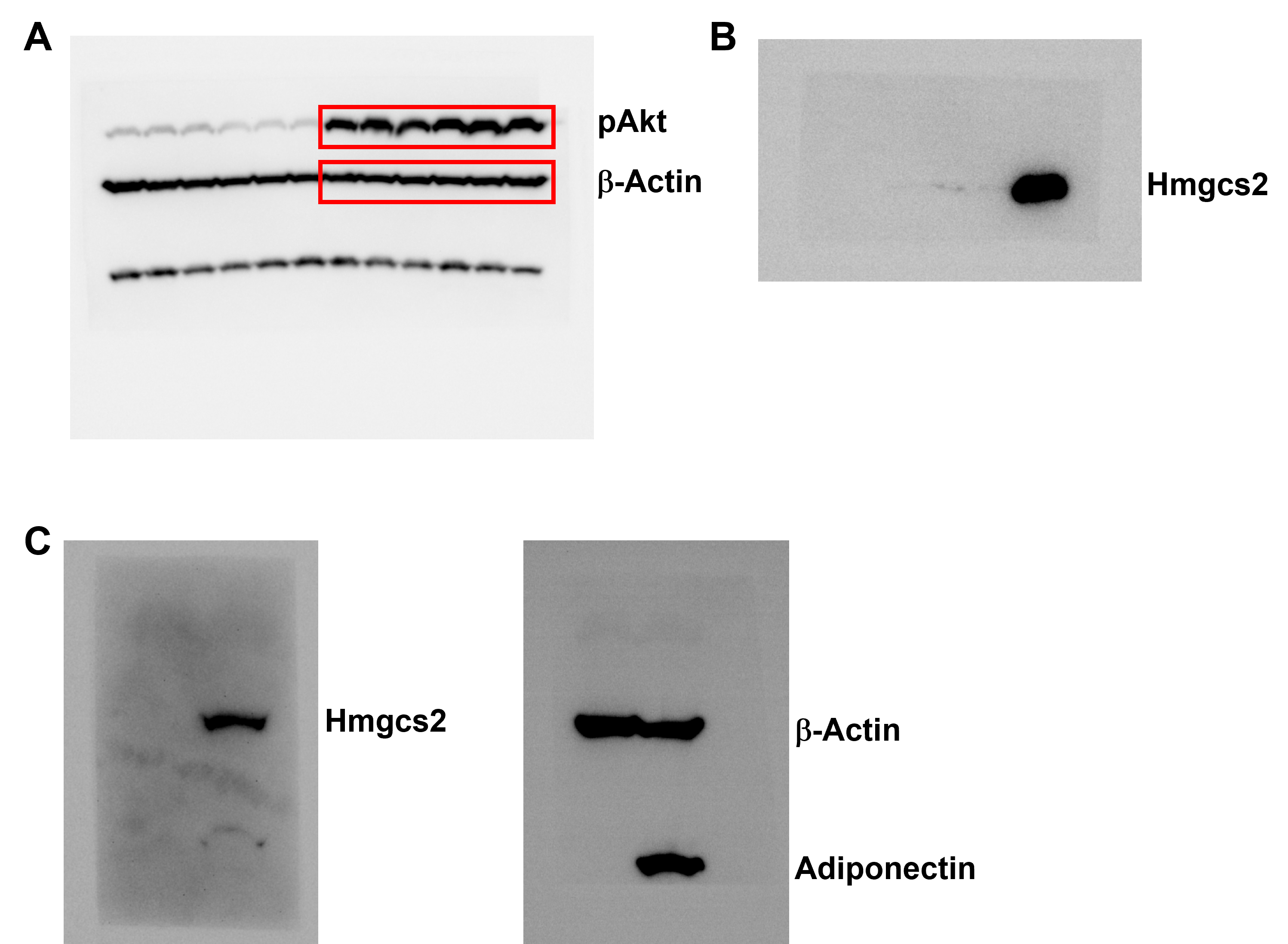


**B**


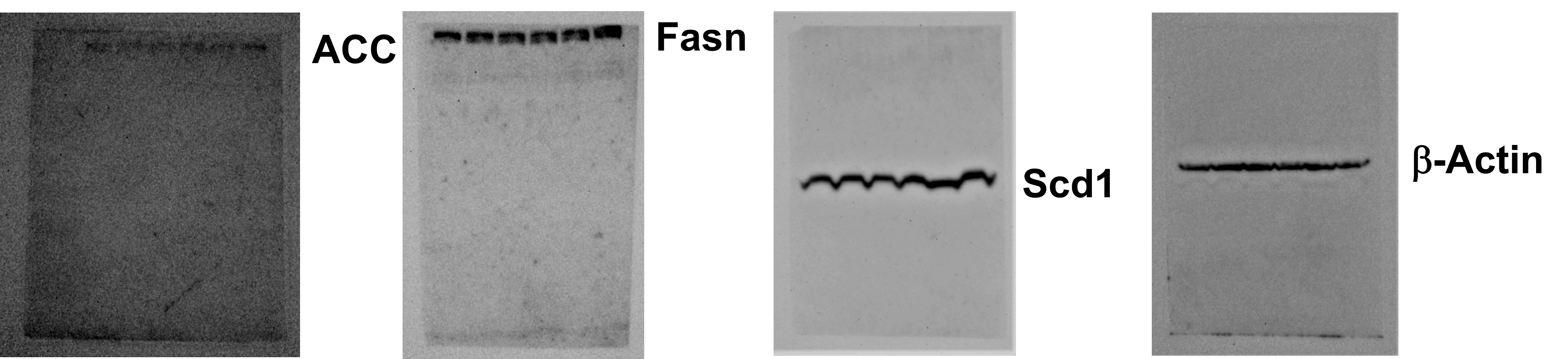


**C**


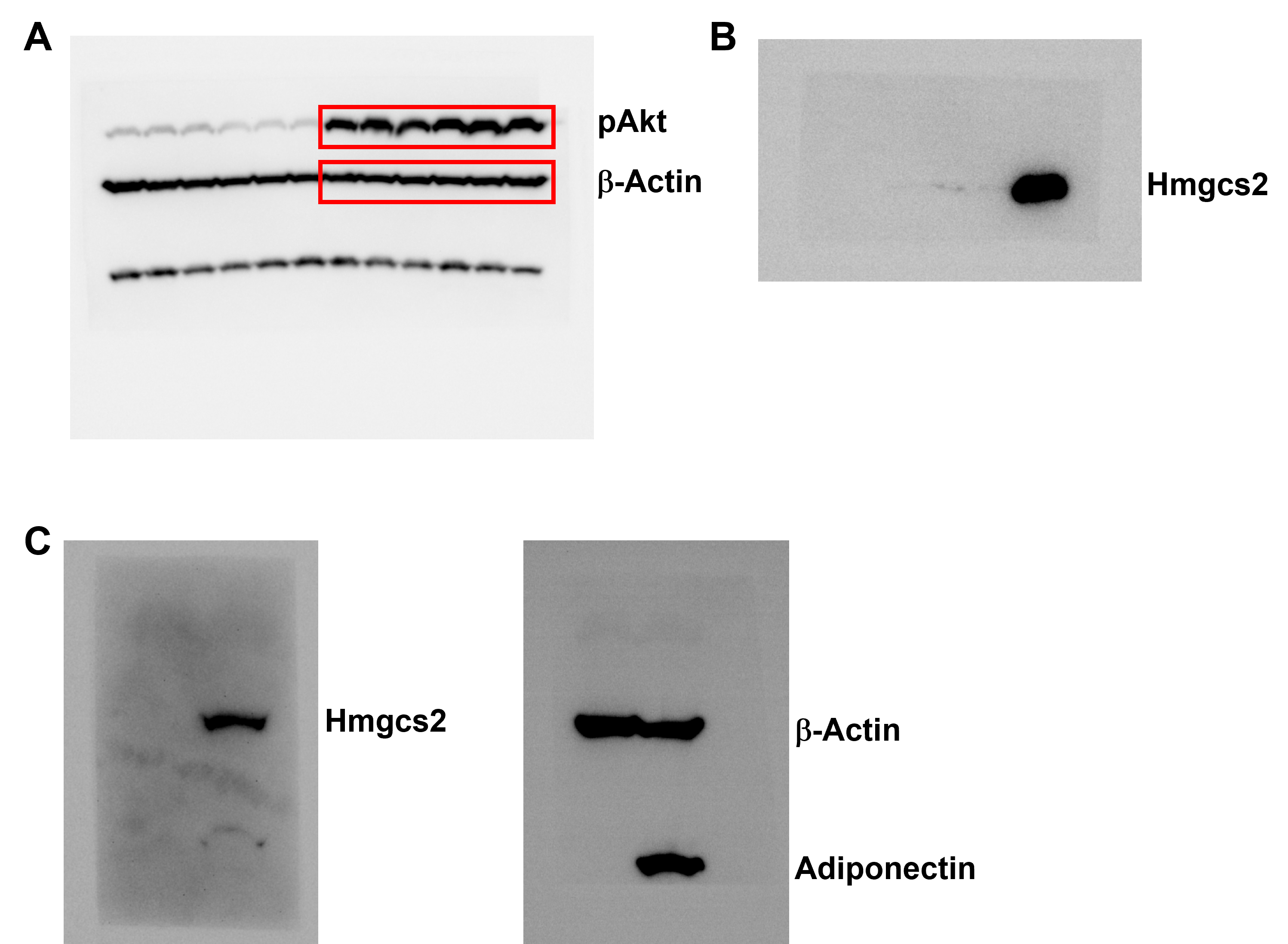


**D**


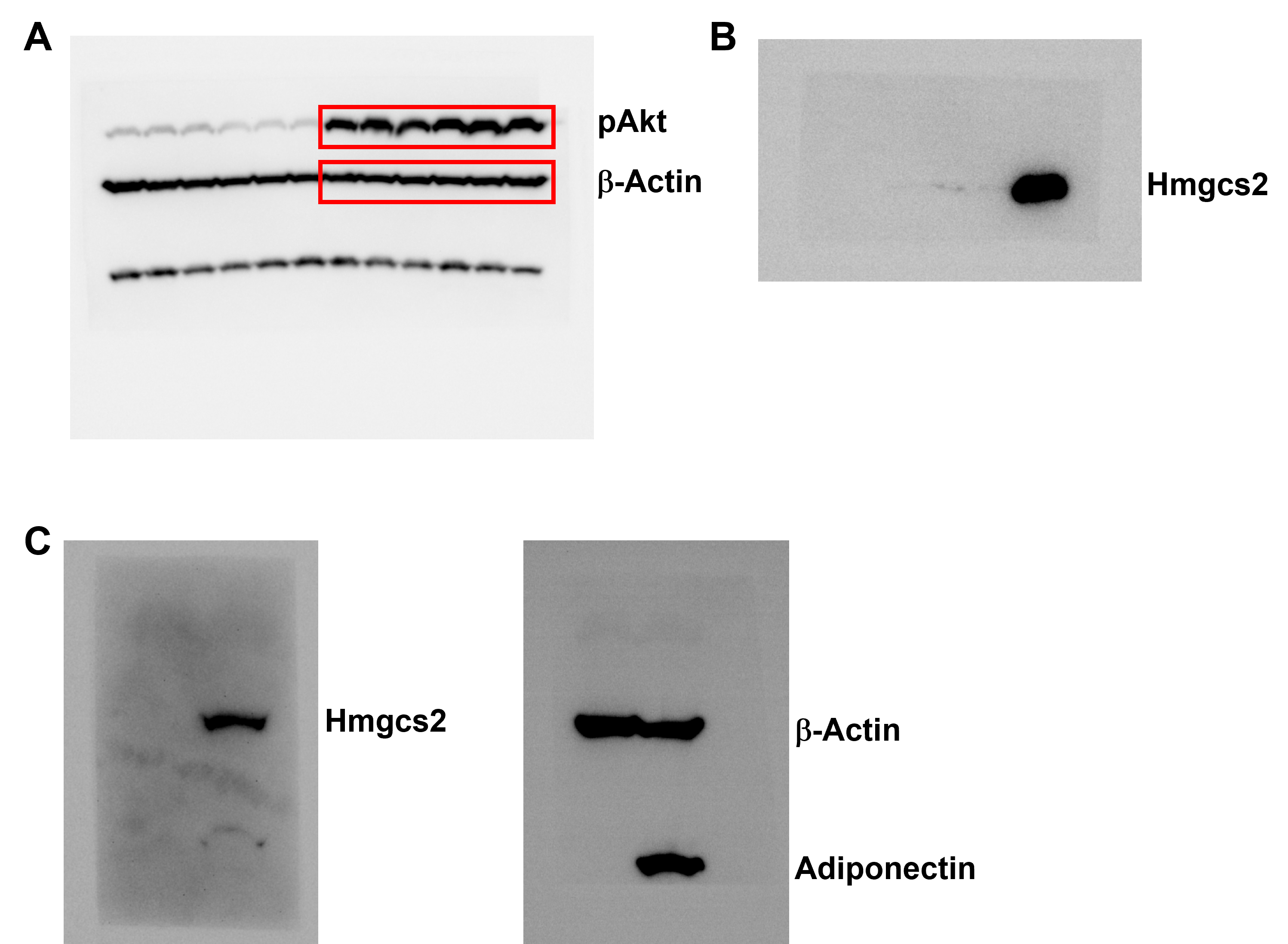


**Figure S4. Pictures are full-length of western blots shown in figure 4, 5, and 6.** (A) fig.4j, (B) fig.4q, (C) fig.5a, and (D) fig. 6g. Membranes are cut prior to hybridization with antibodies, so these are not images of full-length blots. Red line boxes indicate the cropped regions that are presented in the figures.

**Table S1**

| Gene | |  | Sequence (5’ to 3’) |
| --- | --- | --- | --- |
| Hmgcs2 | | Forward | ACCACCAACGCCTGTTATGG |
|  | | Reverse | GCATAGCGACCATCCCAGT |
| Foxo3 | | Forward | AAACGGCTCACTTTGTCCCA |
|  | | Reverse | ATTCTGAACGCGCATGAAGC |
| Mt2 | | Forward | TGTACTTCCTGCAAGAAAAGCTG |
|  | | Reverse | ACTTGTCGGAAGCCTCTTTG |
| SOD1 | | Forward | CAGCATGGGTTCCACGTCCA |
|  | | Reverse | CACATTGGCCACACCGTCCT |
| SOD2 | | Forward | ACGCGCAGATCATGCAGCTG |
|  | | Reverse | GTTCTCCACCACCCTTAGGG |
| Catalase | | Forward | CCAGCGACCAGATGAAGCAG |
|  | | Reverse | CCACTCTCTCAGGAATCCGC |
| PPARγ | | Forward | ATCTTAACTGCCGGATCCACAA |
|  | | Reverse | GCCCAAACCTGATGGCATT |
| Adiponectin | Forward | | GTTCTACTGCAACATTCCGG |
|  | Reverse | | TACACCTGGAGCCAGACTTG |
| Srebp1a | | Forward | GCGCCATGGACGAGCTG |
|  | | Reverse | TTGGCACCTGGGCTGCT |
| Acly | | Forward | ACCCTTTCACTGGGGATCACA |
|  | | Reverse | GACAGGGATCAGGATTTCCTTG |
| ACC | | Forward | GAGGTACCGAAGTGGCATCC |
|  | | Reverse | GTGACCTGAGCGTGGGAGAA |
| Fasn | | Forward | GAGAAGCCATGTGGGGAAGATTTC |
|  | | Reverse | TGAGCAGGGACAGGACAAGAC |
| Scd-1 | | Forward | TGGGTTGGCTGCTTGTG |
|  | | Reverse | GCGTGGGCAGGATGAAG |
| 36B4 | | Forward | GCTCCAAGCAGATGCAGCA |
|  | | Reverse | CCGGATGTGAGGCAGCAG |

**Table S1. qPCR primer sequences, Related to Figures 1, 2h-j, 3-6.**

Hmgcs2, 3-hydroxy-3-methylglutaryl-CoA synthetase 2; Foxo3, Forkhead box O3; Mt2, Metallothionein 2; SOD1, superoxide dismutase 1; SOD2, superoxide dismutase 2; PPARγ, peroxisome proliferative activated receptor gamma; Srebp1a, sterol regulatory element binding transcription protein 1; Acly, adenosine triphosphate citrate lyase; ACC, acetyl-Coenzyme A carboxylase alpha; Fasn, fatty acid synthase; Scd-1, stearoyl-Coenzyme A desaturase 1; 36B4, ribosomal protein, large, P0 (Rplp0)

**Table S2**

| Gene |  | Sequence (5’ to 3’) |
| --- | --- | --- |
| Hmgcs2 | Forward | GGCTGATGGAACGCACAAAG |
|  | Reverse | ATTGAAGAGGGAGGCTGTGC |

**Table S2: qPCR primer sequences, Related to Figure 2a.**

Hmgcs2, 3-hydroxy-3-methylglutaryl-CoA synthetase 2
